# Capsaicin induces apoptosis in MG63 human osteosarcoma cells via the caspase cascade and the antioxidant enzyme system

**DOI:** 10.3892/mmr.2013.1737

**Published:** 2013-10-17

**Authors:** WON HO CHO, HYUN JOO LEE, YOON JI CHOI, JOO HAN OH, HAN SOO KIM, HWAN SEONG CHO

**Affiliations:** 1 Department of Neurosurgery and Medical Research Institute, Pusan National University Hospital, Pusan National University School of Medicine, Busan 602739, Republic of Korea; 2 Department of Orthopaedic Surgery, Seoul National University Bundang Hospital, Gyeonnggido 463707, Republic of Korea; 3 Department of Orthopaedic Surgery, Seoul National University Hospital, Seoul 110744, Republic of Korea; 4 Seoul National University College of Medicine, Seoul 110744, Republic of Korea

**Keywords:** capsaicin, MG63 human osteosarcoma cell, apoptosis, caspase cascade, antioxidant enzyme system

## Abstract

Osteosarcoma is the most common malignant bone tumor in children and adolescents. This aggressive cancer mostly occurs in the long bones. Therefore, novel therapeutic approaches, such as biological therapies and gene therapy, are required to efficiently treat osteosarcoma. Capsaicin (trans-8-methyl-N-vanillyl-6-nonenamide) has been demonstrated to inhibit the growth of several types of cancer cells and a number of studies have shown that osteosarcoma may be vulnerable to biological therapies. However, little is known regarding the therapeutic effects of capsaicin on osteosarcoma. This study investigated the effects of capsaicin on MG63 human osteosarcoma cells, in addition to elucidating the regulatory signaling pathways underlying the effects of capsaicin, the caspase cascade and the antioxidant enzyme system. The MG63 cell line was treated with various concentrations of capsaicin. Cells were analyzed using MTT and flow cytometry, and the presence of DNA fragmentation was evaluated using TUNEL assay. Results showed capsaicin induced apoptosis in MG63 cells. Thus, capsaicin exhibited an anticancer effect in osteosarcoma cells.

## Introduction

Previous studies focused on developing novel reagents that exhibit anticarcinogenic and antimutagenic properties in a number of types of cancer (1–3). Capsaicin (trans-8- methyl-N-vanillyl-6-nonenamide) is an important pungent ingredient with a spicy flavor that is widely used, and may be extracted from chili peppers of the genus *Capsicum*(4,5). It has been demonstrated that capsaicin is able to inhibit the growth of various types of cancer cells, such as human hepatoma carcinoma (6), human colon cancer (7), human breast cancer (8) and human neuroblastoma (9) cells.

Osteosarcoma is the most common malignant bone tumor in children and adolescents (10). This aggressive cancer mostly occurs in the long bones. For the past two decades, chemotherapy and surgery have been commonly used as therapies to improve the condition of patients with osteosarcoma. However, the major problems associated with such intense chemotherapies have increased, with a number of patients showing no improvement in their condition, as a result of the development of resistance against the treatment, and some even presenting with serious side effects in other organs of the body (11–14). Accordingly, novel therapeutic approaches, such as biological therapies and gene therapy, are required to efficiently treat osteosarcoma.

A number of studies have shown that osteosarcoma may be vulnerable to biological therapies (15–17); however, little is known with regard to the therapeutic effects of capsaicin on osteosarcoma. This study examined the effects of capsaicin on MG63 human osteosarcoma cells using 3-(4,5-dimethylthiazol-2-yl)-2,5-diphenyltetrazolium bromide (MTT) assay, flow cytometry, western blot analysis and terminal deoxynucleotidyltransferase-mediated deoxyuridine triphosphate (dUTP) nick end-labelling (TUNEL) assay. In addition, the study explored the regulatory signaling pathway underlying the effects of capsaicin, using a variety of inhibitors.

## Materials and methods

### Reagents

Capsaicin and MTT were purchased from Sigma-Aldrich (St. Louis, MO, USA), while Dulbecco's modified Eagle's medium (DMEM), phosphate-buffered saline (PBS) and fetal bovine serum (FBS) were obtained from Invitrogen Life Technologies (Carlsbad, CA, USA). The U0126, PD98053, SP600125 and Z-VAD-FMK used in the study were purchased from Calbiochem^®^ (Merck KGaA, Darmstadt, Germany) and a chemiluminescence (ECL) kit was obtained from Amersham Pharmacia Biotech (GE Healthcare, Amersham, UK). Bcl-2, Bcl-2-associated X protein (Bax) and pro-caspase-3 were obtained from Epitomics, Inc. (Burlingame, CA, USA), while phosphorylated extracellular signal-regulated kinase (p-ERK), phosphorylated p-38 (p-p38) and phosphorylated c-Jun N-terminal kinase (p-JNK) were purchased from Cell Signaling Technology, Inc. (Beverly, MA, USA).

### Cell line and culture conditions

MG63 cells (human osteosarcoma cell line) were purchased from the Korean Cell Line Bank (Seoul, Korea) and cultured in DMEM containing 10% heat-inactivated FBS. The cells were plated in tissue culture dishes at 37°C in a humidified 5% CO_2_ incubator and cultured for 2–4 days until confluence was reached. Subcultures were prepared using 0.05% trypsin solution and seeded in 6- or 96-well tissue culture plates. Serum was starved from the culture media at the time of adding various agents.

### Measurement of cell growth by MTT

Cell viability was assessed using an MTT assay, based on the reduction of MTT into formazan dye by the action of mitochondrial enzymes. MG63 cells were seeded in 96-well plates at a density of 5×10^2^ cells per well and indicated concentrations of capsaicin were added for indicated time-periods. Briefly, following treatment with capsaicin at various concentrations (0, 50, 100, 150, 200, 250 and 400 μM) and various time-periods (0, 3, 6, 12, 24 and 48 h) under 150 μM of capsaicin, the cells were washed and 0.5 mg/ml MTT in DMEM solution was added to each well, prior to incubation for 2 h at 37°C. The supernatant was then removed and the cells were dissolved in dimethylsulfoxide (DMSO). The absorbance of each well was measured at 570 nm with a 680 microplate enzyme-linked immunosorbent assay (ELISA) reader (Bio-Rad Laboratories, Hemel Hempstead, UK).

### Cell morphology

The untreated and treated cells were seeded in 6-well plates at a density of 5×10^4^ cells per well and incubated for 24 h with 50–400 μM capsaicin (0, 50, 100, 150, 200, 250 and 400 μM). Cell morphology was examined under a light microscope.

### Flow cytometric analysis

Cells were seeded in 6-well plates at a density of 5×10^4^ cells per well and treated with the indicated reagents for 24 h at 37°C. The suspended and adherent cells were then harvested using 0.05% trypsin solution. The harvested cells were centrifuged at 10,000 × g for 15 min at 4°C and the pellets were then washed in PBS, prior to the addition of fixing solution with ice-cold 100% ethanol containing 0.25% Triton X-100 for treatment overnight at 4°C. Subsequent to fixation, the cells were washed and stained with 50 μg/ml propidium iodide containing 100 μg/ml RNase, prior to being incubated for 20 min at 37°C and analyzed using a FACSort flow cytometer (Becton Dickinson, Franklin Lakes, NJ, USA).

### TUNEL assay

The presence of DNA fragmentation was evaluated using TUNEL assay with an *in situ* Cell Death Detection kit (fluorescein) from Roche Applied Science (Indianapolis, IN, USA). The cells were seeded in cover slides (5×10^2^ cells per slide) and then treated with capsaicin. Following this, the cells were washed in PBS and freshly prepared 4% paraformaldehyde was added for cell fixation for 1 h at 37°C in a humidified 5% CO_2_ incubator. The cells were then washed again in PBS, prior to being permeabilized in permeabilization solution (0.1% Triton X-100 in 0.1% sodium citrate) for 2 min on ice. The cells were then subjected to the TUNEL reaction at 37°C in a humidified atmosphere in the dark for 60 min. The fluorescence signal emitted by the fluorescein-labeled dUTP incorporated into the fragmented DNA was detected using Leica confocal microscopy (Leica Microsystems, Wetzlar, Germany).

### Measurement of cell death using the trypan blue dye exclusion assay

Capsaicin-treated cells were harvested using 0.05% trypsin solution and then suspended with 0.4% trypan blue solution. The cells were counted using a hemocytometer under a light microscope and cells that were observed to exclude the dye were considered viable.

### Western blot analysis

The cells were seeded in 6-well plates at a density of 5×10^4^ cells/cm^2^, cultured and incubated in DMEM containing 10% FBS. Prior to treatment with the indicated conditions, the cells were serum-starved overnight, treated with the agent and then harvested. Using lysis buffer [20 mM Tris-HCl (pH 7.4), 10 mM NaCl, 1 mM EDTA, 1 mM ethylene glycol-O-O′-bis(2-amino-ethyl)-N,N,N′,N′-tetraacetic acid (EGTA), 0.1 mM phenylmethylsulfonyl fluoride, protease inhibitor cocktail and 1% Triton X-100], the cells were lysed on ice. The lysates were subsequently centrifuged at 10,000 × g for 20 min at 4°C and the supernatants were loaded on 15% sodium dodecyl sulphate-polyacrylamide gel electrophoresis gel and transferred to a nitrocellulose membrane. The membranes were subsequently immunoblotted with various primary antibodies and incubated with the respective peroxidase-conjugated secondary antibodies. The signals were visualized using an enhanced ECL kit from Amersham Pharmacia Biotech.

### Statistical analysis

Experiments were performed at least three times. Statistical significance was analyzed using a Student's t-test (two-tailed). P<0.05 was considered to indicate a statistically significant difference.

## Results

### Inhibitory effects of capsaicin on the cell viability of osteosarcoma cells

**Figure 1:**
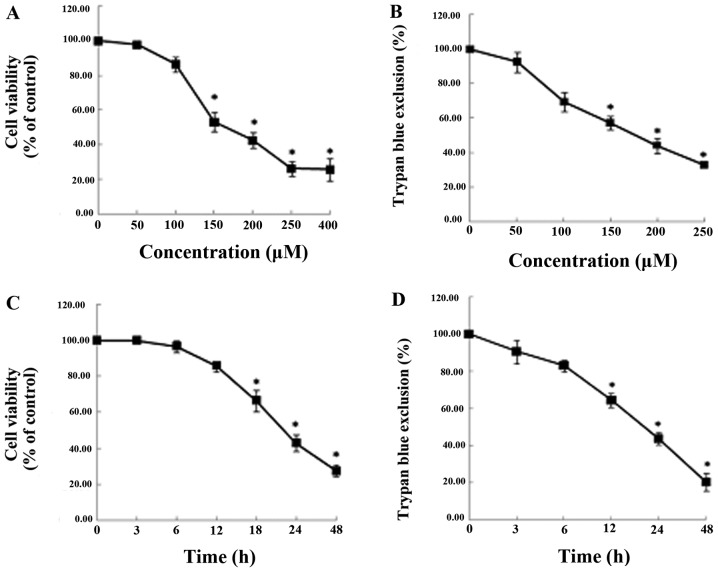
Capsaicin-induced loss of cell viability in MG63 cells. MG63 cells were treated with the indicated concentrations of capsaicin for 24 h and with 150 μM capsaicin for the indicated time-periods. The cells were analyzed using (A and C) MTT assay, and (B and D) trypsin blue exclusion assay. The data reported are the mean ± SEM of four independent experiments. ^*^P<0.05 compared with the control without capsaicin.

We examined the effects of capsaicin in the MG63 osteosarcoma cell lines, which had been treated with various concentrations of capsaicin (0, 50, 100, 150, 200, 250 and 400 μM) for various time-periods (0, 3, 6, 12, 24 and 48 h). As shown in Fig. 1, capsaicin reduced the viability of the MG63 cells in a dose- and time-dependent manner, as demonstrated using MTT (Fig. 1A and C) and trypan blue exclusion (Fig. 1B and D) assays. The viability of the cells treated with 150 μM capsaicin for 24 h was markedly reduced.

### MG63 cell morphology observed using light microscopy

**Figure 2:**
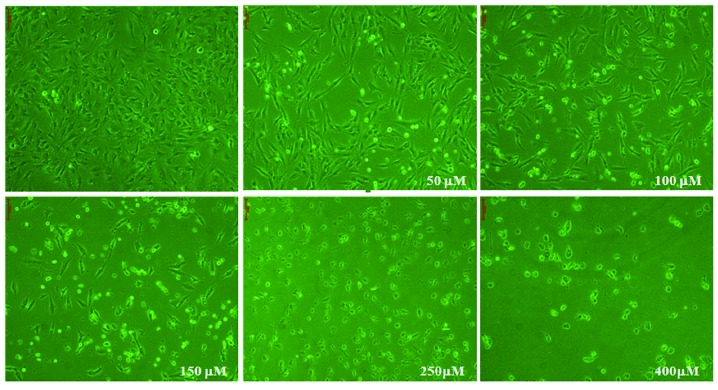
Cell morphology of MG63 cells with different concentrations of capsaicin treatment. MG63 cells were left untreated or treated with capsaicin at concentrations of 50, 100, 150, 250 and 400 μM for 24 h. Scale bar, 200 μm.

MG63 cells were cultured for 24 h with different concentrations of capsaicin (0, 50, 100, 150, 250 and 400 μM). Following 24 h treatment with capsaicin, no significant morphological changes were observed in the cells treated with capsaicin at 50 and 100 μM. However, the cells exhibited the morphological features of apoptosis when treated with 150 μM capsaicin for 24 h (Fig. 2). These morphological changes of the cells represented the apoptotic cell death that occurred with 150 μM capsaicin at the end of the 24-h exposure.

### Capsaicin-induced apoptosis in MG63 cells

**Figure 3:**
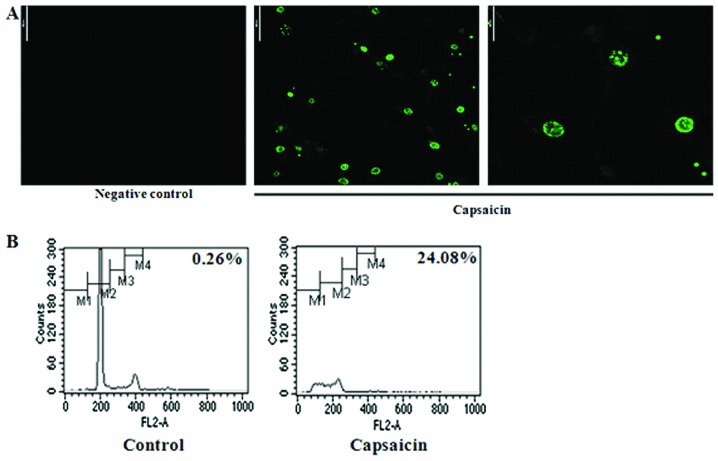
Induction of apoptosis in MG63 cells by capsaicin. (A) DNA fragmentation of MG63 cells treated with 150 μM capsaicin for 24 h was detected using terminal deoxynucleotidyltransferase-mediated deoxyuridine triphosphate (dUTP) nick end-labelling (TUNEL) assay. (B) The DNA fragmentation was characterized as capsaicin-induced apoptotic cell death using flow cytometric analysis. Scale bar, 400 μm. The percentage of the sub-G1 peak is indicated by M1.

In order to characterize the type of cell death that had been observed, we examined whether the cell death was apoptosis. MG63 cells were treated with 150 μM capsaicin for 24 h and the apoptotic DNA fragmentation of the MG63 cells was visualized using TUNEL assay. TUNEL assay is a tool that is frequently used for the detection of DNA fragmentation (18,19). The significant increase in the number of TUNEL-positive cells (green) showed that capsaicin induced apoptosis in the MG63 cells (Fig. 3A). In addition, as shown in Fig. 3B, capsaicin treatment resulted in an increased proportion of cells in the G0–G1 phase, from 0.26 to 24.8%. The G0–G1 phase is an indicator for cell apoptosis when increased. These results suggested that capsaicin induced apoptosis.

**Figure 4:**
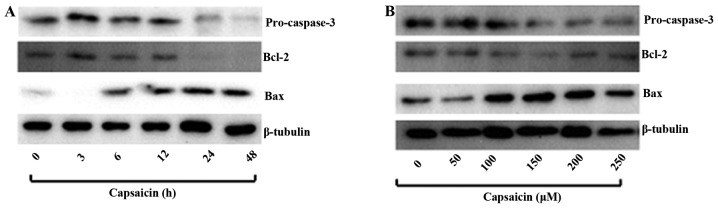
Effects of capsaicin on the MG63 cells and the protein molecules involved in apoptosis. Pro-caspase-3, Bcl-2 and Bcl-2-associated X protein (Bax) were expressed in a (A) dose- and (B) time-dependent manner, as shown using western blot analysis. The loading control was β-tubulin.

To investigate the effect of capsaicin on protein molecules that are involved in apoptosis, western blot analysis was used to test for the presence of the anti-apoptotic proteins Bcl-2 and cleaved caspase 3 (pro-caspase-3) and the pro-apoptotic protein Bax. MG63 cells were treated with various concentrations of capsaicin for 24 h and with 150 μM capsaicin for different time-periods. Capsaicin decreased the expression of pro-caspase-3 and Bcl-2, while the expression of Bax was increased in a dose- and time-dependent manner (Fig. 4A and B).

### Identifying the signaling pathway that regulates the capsaicin-induced cell death

**Figure 5:**
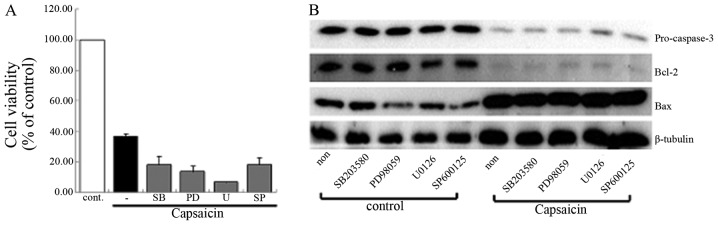
Involvement of mitogen-activated protein kinase (MAPK) subfamilies in the effects of capsaicin on the MG63 cells. The effects of MAPK inhibitors (pre-treatment with 20 μM SB203580, PD98059, U0126 and SP600125, respectively, for 60 min) on MG63 cells treated with 150 μM capsaicin for 24 h were analyzed using (A) MTT assay and (B) western blot analysis. Cont, control; SB, SB203580; PD, PD98059; U, U0126; SP, SP600125.

**Figure 6:**
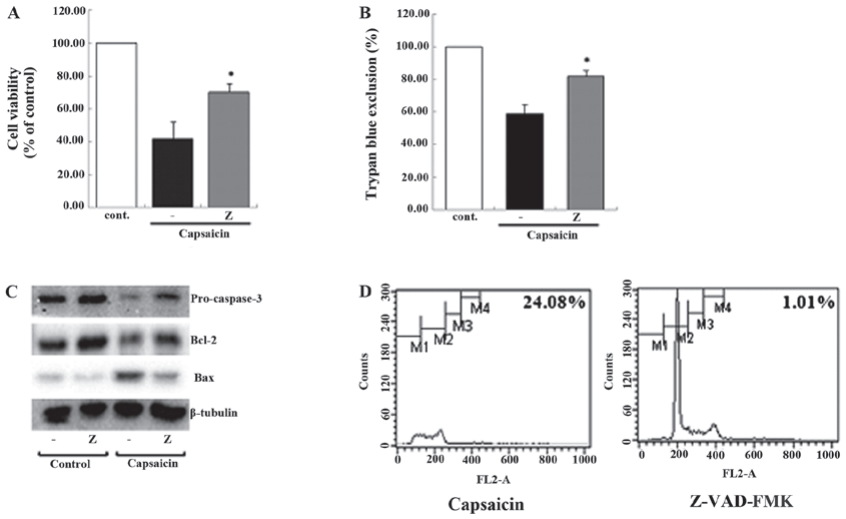
Involvement of caspase in the effects of capsaicin on the MG63 cells. The effects of a caspase general inhibitor (pretreatment with 20 μM Z-VAD-FMK for 60 min) on MG63 cells treated with 150 μM capsaicin for 24 h were analyzed using (A) MTT assay, (B) trypan blue exclusion assay, (C) western blot analysis and (D) flow cytometric analysis. The percentage of the sub-G1 peak is indicated by M1. Cont, control; Z, Z-VAD-FMK. The data reported are the mean ± SEM of four independent experiments. ^*^P<0.05 compared with the control.

**Figure 7:**
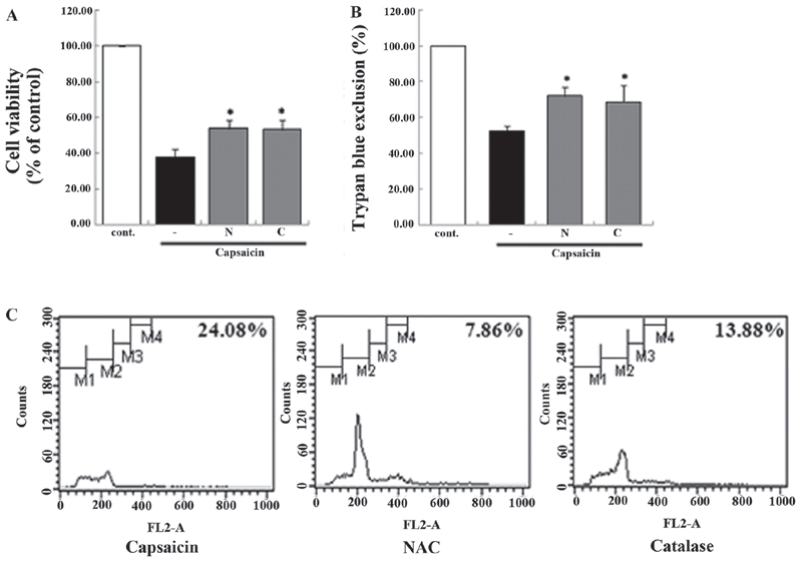
Involvement of antioxidant enzymes in the effects of capsaicin on the MG63 cells. The effects of 10 μM N-acetyl-L-cysteine (NAC) for 90 min and 800 U/ml catalase for 90 min on MG63 cells treated with 150 μM capsaicin for 24 h were analyzed using (A) MTT assay, (B) trypan blue exclusion assay and (C) flow cytometric analysis. The percentage of the sub-G1 peak is indicated by M1. Cont, control; N, NAC; C, catalase. The data reported are the mean ± SEM of four independent experiments. ^*^P<0.05 compared with the control.

It has been demonstrated that apoptosis leads to various signaling processes and, among them, the mitogen-activated protein kinases (MAPKs) (20), the caspase cascade (21) and the antioxidant enzyme system (22) are the major executors of the process of apoptosis. We initially suggested that the MAPK signaling pathway was involved in the capsaicin-induced apoptosis. However, the group that was pretreated with MAPK inhibitor did not show any differences when compared with the group treated only with capsaicin using MTT assay and western blot analysis (Fig. 5). The data demonstrated that capsaicin-induced apoptosis was not regulated by the MAPK signaling pathway in MG63 cells. The involvement of the caspase cascade in the capsaicin-induced apoptosis using MTT assay, trypan blue exclusion, western blot analysis and flow cytometry was then examined. Consistently, it was shown that the general caspase cascade inhibitor, Z-VAD-FMK, had some effect when the results were compared with those from the group treated only with capsaicin (Fig. 6). These results suggested that the caspase cascade was involved in capsaicin-induced apoptosis. In addition, the antioxidant enzyme system was demonstrated to be involved in the capsaicin-induced apoptosis. In the groups that were pretreated with antioxidant enzyme inhibitor, the viability of the MG63 cells decreased from 24.08 to 7.86% [N-acetyl-L-cysteine (NAC)] and 13.88% (catalase) compared with the group treated only with capsaicin. This result showed that antioxidant enzyme inhibitors affected the apoptosis using a variety of methods (Fig. 7). We demonstrated that antioxidant enzymes were involved in the capsaicin-induced apoptosis in MG63 cells.

## Discussion

In this study, we used a variety of techniques and demonstrated that the cell viability of MG63 cells was able to be reduced by capsaicin in a dose- and time-dependent manner. In addition, the capsaicin-treated group showed an increased level of positivity with the TUNEL assay and an increase in Bax protein. Moreover, the experiments with the signaling pathway inhibitors showed that the groups pretreated with Z-VAD-FMK, NAC and catalase, respectively, had different results compared with those from the group treated only with capsaicin. These results indicated that the capsaicin-induced apoptosis in MG63 cells may have been mediated by the caspase cascade and the antioxidant enzyme system, among various signaling pathways.

Osteosarcoma is the most frequently occurring primary malignant neoplasms of the long bones, including the distal femur and the proximal humerus, and mainly affects children and adolescents (10,23). The prognosis for osteosarcoma, for which conventional treatments include surgery, chemotherapy and radiotherapy, is poor due to the early pulmonary metastasis and limited improvements. Chemotherapy has become a foundation for the basic treatment of osteosarcoma. A number of studies have focused on the development of new effective therapeutic strategies for osteosarcoma, using novel materials extracted from natural food substances that exhibit an anticancer effect, despite the successful use of neoadjuvant chemotherapy in the treatment of osteosarcoma (11,12,15,24).

It has been demonstrated that a number of reagents are able to induce apoptosis on MG63 human osteosarcoma cells (25–27); however, the effect of capsaicin on MG63 cells has remained unclear. Capsaicin, the main pungent ingredient in the genus *Capsicum*, has long been used in drugs for weight loss and has been studied as an attractive drug for cancer treatment, as an agent that induces apoptosis in various cell types *in vitro*(28–31). Moreover, the compound has been indicated to promote apoptosis *in vivo* as the mechanism of tumor cell elimination in animal models for carcinogenesis (32,33). These observations have continued to draw attention to capsaicin as a possible anticancer agent.

Apoptosis has been suggested as a promising target for cancer chemotherapy (34–36). Apoptosis is a form of self-regulated cell death and occurs during normal cell turnover, development and immune regulation (37,38). The characteristic morphological changes involved in apoptosis include cytoplasmic shrinkage, plasma membrane blebbing, chromatin condensation and the formation of apoptotic bodies containing well-preserved organelles. In addition, during apoptosis cells undergo double-strand cleavage of nuclear DNA (34,39–41).

This study was designed to investigate capsaicin-induced apoptosis in MG63 human osteosarcoma cells and its underlying molecular mechanisms. Using TUNEL assay, flow cytometric assay and western blot analysis, we demonstrated that the anticancer effect of capsaicin resulted in morphological changes, decreased cell viability and apoptosis in the MG63 cells (Figs. 1–4). These results showed that capsaicin was able to inhibit cell viability and growth and induce apoptosis.

We investigated the molecular factors that were involved in the apoptosis of capsaicin-treated MG63 cells. The MAPKs are expressed in all mammalian cell types and have individually different functions in the regulation of specific cell responses. MAPKs have been demonstrated to be composed of three parallel kinase modules, including ERK, JNK and p-38-MAPK (42–44). As shown in numerous studies, the MAPK signaling pathway is important in the regulation of cellular growth, differentiation, survival, angiogenesis and apoptosis (20,45,46–48). Accordingly, we initially suggested that the MAPK signaling pathway was involved in the cellular response of capsaicin-induced apoptosis. Using groups pretreated with MAPK inhibitors, it was revealed that MAPKs exerted no specific effect in capsaicin-induced apoptosis in the MG63 cells (Fig. 5).

It has been revealed that caspase, or cysteine-aspartic protease, belongs to the group of enzymes known as cysteine proteases, which are homologous to the *Caenorhabditis elegans*, the cell death gene, CED-3 (21,49). Cysteine proteases have multi-faceted functions in virtually every aspect of physiology, such as in growth and development, senescence and apoptosis (50,51). Moreover, the components of the caspase cascade are present in various cells in the form of inactive zymogens, which are then activated to convey the apoptotic signal (52). Furthermore, it has been suggested that the caspase cascade may induce the apoptotic reaction (53). Our results showed that the caspase cascade regulated capsaicin-induced apoptosis, observed through cell viability, western blot analysis and flow cytometry (Fig. 6).

In present study it was demonstrated that the antioxidant enzyme system was also involved in the capsaicin-induced apoptosis. The antioxidant enzyme system has been indicated to be important in the control of apoptosis (54,55). In addition, antioxidant enzymes defend cells from oxidative damage, such as reactive oxygen species (ROS) production (56–58). ROS interact with a wide range of cell components and cause damage to cell structures, including the membrane, and are regulated with apoptosis (30,59–61). As such, antioxidant enzymes have the potential to protect the cells from oxidative damage. Based on the results of our study, we verified that the antioxidant enzyme system was particularly effective in capsaicin-induced apoptosis in the MG63 cells, as demonstrated using a variety of methods (Fig. 7). Therefore, it was indicated that ROS were part of the capsaicin-induced apoptosis pathway in the MG63 cells.

The present study provided distinct results describing the effect of capsaicin on MG63 cells, in addition to elucidating the molecular mechanisms that were implicated in the indution of apoptosis. In combination, the results showed that capsaicin induced apoptosis in the MG63 cells and that the caspase cascade and antioxidant enzyme system were the underlying regulatory signaling pathways involved in the capsaicin-induced apoptosis. In a previous study, we demonstrated the effect of capsaicin on human glioblastoma U87MG cells and concluded that capsaicin induced apoptosis in the U87MG cells (62). The present results indicated that capsaicin exhibited an anticancer effect in osteosarcoma cells. Further *in vitro* and *in vivo* studies are required before capasaicin is able to be ultimately applied to treat human patients with osteosarcoma.
